# Blooming Urban Table: Flower Resources for Butterflies in Small Wastelands of a Large European City

**DOI:** 10.1002/ece3.72088

**Published:** 2025-09-15

**Authors:** Sylwia Pietrzak, Krzysztof Pabis

**Affiliations:** ^1^ Department of Invertebrate Zoology and Hydrobiology University of Lodz Lodz Poland

**Keywords:** biodiversity, flowering plants, lepidoptera, urbanisation

## Abstract

Insect–flower relationships are among the most important ecological interactions occurring in the terrestrial ecosystem. In urban areas, such interactions are often disturbed or altered. However, few studies report floral affinities of butterflies in Europe, and our understanding of flower utilisation by butterflies in urban habitats is limited. Our study included long‐term (2017–2022), qualitative observations and quantitative analyses of flower visits performed in 2021 and 2022 on five urban wastelands located in Łódź (Central Poland). We have recorded observations of 39 butterfly species on 81 species of plants (representing 19 families and 16 orders of plants). The majority of butterfly species were associated with many species of plants. We did not record tight associations with the colour of flower or the depth of flower, although in general, butterflies were observed on pink (21 species of plants), yellow (20 species of plants), white (17 species of plants) and violet (13 species of plants) flowers, while they were not recorded on orange, blue or red flowers. The most commonly visited flowering plants represented Asteraceae, Fabaceae and Lamiaceae. The highest number of butterflies was recorded on *Jasione montana* (22 species of butterflies), *Cirsium arvense* (19 species of butterflies), *Berteroa incana* (22 species of butterflies), *Trifolium pratense* (22 species of butterflies) and *Origanum vulgare* (21 species of butterflies). The highest number of individuals was observed on *Centaurea stoebe* , *Senecio jacobaea* , *Cirsium arvense* and *Echium vulgare* . Our study showed that urban wastelands provide flower resources for butterflies and should be included in the management practices of large cities.

## Graphical abstract

There are almost no studies of butterfly flower preferences in urban habitats, and overall knowledge about their floral affinities in Europe is very scarce. Our study was based on long‐term (2017–2022) qualitative observations and quantitative studies of flower visits performed in 2021 and 2022 on several urban wastelands. The results may have importance for urban ecologists, entomologists and city management practice.

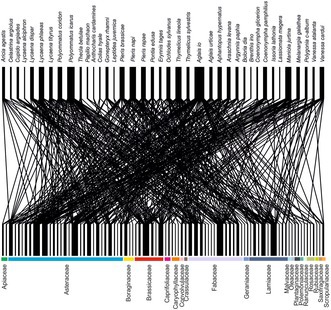

## 1 Introduction

Butterflies are important pollinators, although not as efficient as bees and other hymenopterans (Barrios et al. 2016; Ollerton 2017); however, sometimes their effectiveness is comparable to bees (Cusser et al. 2021; Ollerton et al. 2025). The long‐term angiosperm‐lepidopteran coevolution resulted in a diversity of adaptations associated with the utilisation of flower resources (Asar et al. 2022; Peris and Condamine 2024). All butterflies have well‐developed proboscis and are mainly nectar feeders that reflect the full spectrum of flower specialisation (Kristensen et al. 2007; Jain et al. 2016). They may favour particular components of nectar (Alm et al. 1990; Erhardt 1991), display flower colour preferences (Pohl et al. 2011; Arikawa 2017) and can learn how to associate flower colour with nectar reward (Drewniak et al. 2020). Some butterflies are dependent on single species of plants (Jain et al. 2016), while others forage on a large number of species (Martinez‐Adriano et al. 2018). Their floral affinities may or may not match the host‐plant preferences of caterpillars (Menken et al. 2010; Tudor et al. 2004; Altermatt and Pearse 2011). The length of the proboscis may also differ among species, allowing for the penetration of flowers of different shapes, depths and sizes. Sometimes, butterflies do not pollinate flowers, resulting in so‐called ‘nectar robbery’ (Bauder et al. 2015). Moreover, differences in the length of the proboscis can be found between individuals of the same species, resulting in different flower preferences (Szigeti et al. 2020). In general, pollinator attraction to flowers is mediated by multimodal signals that are still relatively scarcely studied and poorly understood (Pohl et al. 2011; Erickson, Grozinger, and Patch 2022; Erickson, Junker, et al. 2022).

Flower visits can be affected by the presence of predators (Fukano et al. 2016), weather conditions, habitat type and time of season (Primack and Inouye 1993; Mertens et al. 2021). Urbanisation levels (Herrmann et al. 2023) and air pollution may distract the insects by altering floral odours (Ryalls et al. 2022). Natural and anthropogenic changes in plant communities may also alter flower preferences in particular habitats or even influence the distribution of some species (Steffan‐Dewenter and Westphal 2008; Thomas et al. 2011; Curtis et al. 2015; Martinez‐Adriano et al. 2018). Therefore, butterfly–flower associations could be important for conservation strategies, because flower specialists are often species of conservation concern (Tudor et al. 2004).

Even though European butterfly fauna are probably the most comprehensively studied in all possible aspects, our knowledge about the flower preferences of even common butterfly species is still relatively limited (Jennersten 1984; Tudor et al. 2004; Curtis et al. 2015; Shackleton and Ratnieks 2016). Those gaps are even more pronounced in urban areas (e.g., Bergerot et al. 2010; Dylewski et al. 2020; Herrmann et al. 2023). The nourishment in the adult stage may extend lifespan, increase overwintering survival of the adults and enhance the quality and number of eggs, ultimately leading to more successful offspring, and it is one of the key elements of butterfly biology (Mevi‐Schütz and Erhardt 2005; Geister et al. 2008; Cahenzli and Erhardt 2012). The lack of appropriate nectar supply might be a limiting factor of butterfly distribution and a reason for population declines (Curtis et al. 2015). Therefore, we must pay particular attention to flower resources used by butterfly communities in large agglomerations. Urban environments are known for altered ecological interactions (Theodorou 2022), disturbances (Fenoglio et al. 2021), habitat fragmentation and the development of specific plant communities that would not develop in other circumstances (Kühn and Klotz 2006; Wittig and Becker 2010; Lososová et al. 2012b, 2012a; Deák et al. 2016). Flowering plants observed in cities consist of species that may cope well with pollution, water shortage and high temperatures (McKinney 2008; Deák et al. 2016; Kalusová et al. 2017), but do not necessarily constitute rich food resources for pollinators (Tew et al. 2021).

The main focus of earlier studies was directed at pollinator associations with garden plants (Di Mauro et al. 2007; Garbuzov and Ratnieks 2014; Garbuzov, Madsen, and Ratnieks 2015; Garbuzov, Samuelson, and Ratnieks 2015; Shackleton and Ratnieks 2016; Garbuzov et al. 2017; Marquardt et al. 2021) and rarely on spontaneous vegetation (Tiple et al. 2006; Herrmann et al. 2023). At first, this approach seems to be understandable because cultivated ornamental plants are recognised as one of the richest and most diverse sources of nectar (Tew et al. 2021; Plummer et al. 2023). In quantitative studies, it is also methodologically convenient to conduct standardised counts if we can control the number of plots in a garden (Shackleton and Ratnieks 2016). Recent studies demonstrated that other urban habitats like parks, railways, pathways, wastelands and various areas covered by spontaneous vegetation may constitute valuable resources for pollinators (Bonthoux et al. 2019; Dylewski et al. 2019; Twerd and Banaszak‐Cibicka 2019; Theodorou et al. 2020; Pietrzak and Pabis 2024). These neglected communities of native and alien plants might provide a food resource in various types of disturbed and highly modified ecosystems (Ricotta et al. 2012; Rollin et al. 2016).

At the same time, we know almost nothing about the flowering plants utilised by European butterflies in urban areas. Without similar studies, it will be impossible to provide successful and sustainable management practices (Aguilera et al. 2019), urban grassland restorations (Klaus 2013) or the creation of urban flower meadows (Hicks et al. 2016). Studies of urban flower–butterfly relationships are also very important because of global urbanisation trends (Soni et al. 2025). Cities are good models of ecosystem change that may help to make reliable predictions also in other ecosystems affected by human activities (Chen et al. 2014). One of the recent studies suggested that urban wastelands might provide an important refuge for butterflies in the cities surrounded by disturbed agricultural landscape (Pietrzak and Pabis 2024). Similar conclusions were drawn based on studies of bees (Twerd and Banaszak‐Cibicka 2019). It is a paradox that the most highly altered areas might become habitat islands that help to preserve diversity on a larger spatial scale, especially in densely populated areas of Europe. Therefore, it is important to point at the most important resources that could facilitate and maintain butterfly biodiversity in the cities. Our study aims to explore flower resources utilised by butterfly communities associated with wasteland habitats of a large central European city.

## 2 Materials and Methods

### 2.1 Study Area

Łódź is the fourth largest (300 km^2^, 660,000 citizens) city in Poland, and it is located in Central Europe. It is a relatively young city that developed in the 19th century as a result of textile manufacturing development (Markowski et al. 1998; Witosławski 2006). It is uniformly built and not divided by any large rivers. Three urbanisation zones were distinguished in Łódź: inner city (zone I), peri‐urban area (zone II) and outskirts (zone III; Janiszewski et al. 2009). Zones II and III are characterised by a larger number of green spaces, including parks, gardens, wastelands or even agricultural lands.

### 2.2 Field Studies and Data Analysis

The analysis of flower–butterfly associations was performed in parallel to the quantitative analysis of butterfly abundance and diversity based on Pollard walks (Pietrzak and Pabis 2024). Studies were conducted on five large wastelands located in the peri‐urban area (zone II) and outskirts (zone III). Observations occurred weekly from April to September in 2019 and 2020 for a total of 214 visits. Each visit lasted for 4–6 h, allowing for comprehensive data collection. Additionally, all other observations of butterfly–flower associations were recorded between 2017 and 2022 at different locations within the city borders. These five major sampling sites had a surface area of 2–3 ha. All sampling sites were characterised by a mosaic of microhabitats that included dry and moist meadows, as well as patches of shrubs and trees (Pietrzak and Pabis 2024). Our dataset is fully qualitative as the number of visits per flower was not counted, and there was no data about the observations in each month of the year. We were recording every new flowering plant observed as part of the diet of particular butterfly species. Therefore, all analyses are based on presence/absence data. Nevertheless, the results of the Pollard walks constitute a background for this study and provided information about the abundance of all analysed butterfly species in the investigated urban wastelands. All plants were categorised into groups based on the colour of flowers (pink, yellow, white, violet, white/yellow, orange, blue, grey and red), plant growth form (herbaceous plants, shrubs, trees) and depth of flower (shallow, medium, deep). The last division was based on the length of the corolla tube based on the classification for Polish flora (Rutkowski 2004). The diet of each butterfly species and family was analysed by the number of used plants and the species composition of plants used. Analysis of similarity between butterflies was based on the Bray‐Curtis formula using a group‐average method (Clark and Warwick 2001). We have used the presence/absence records of plant species in the diet of each butterfly. This analysis allows us to present the ecological similarities between butterfly species and designate the groups of species that are associated with similar groups of flowering plants. To visualise foodweb relationships between butterfly species and their food plants, the bipartite::plotweb function was used (Dormann et al. 2008).

Quantitative analysis of butterfly visits was also conducted on 10 species of plants, including *Berteroa incana* (white flowers), *Jasione montana* (violet flowers), *Echium vulgare* (blue flowers), *Centaurea jacea* (pink flower), *Centaurea stoebe* (pink flower), *Solidago gigantea* (yellow flower), *Cirsium arvense* (violet flower), *Trifolium pratense* (pink flower), *Origanum vulgare* (pink flower), *Senecio jacobaea* (yellow flower). We have selected common species of plants recorded at investigated sites that were visited by many butterfly species during our qualitative observations conducted in 2019 and 2020. Here, we followed a method proposed for garden plants (Shackleton and Ratnieks 2016) by restricting plots of field‐selected plants. Restricted plots of each plant were selected in the field. Butterflies visiting each plot were counted during 12 visits conducted at regular intervals (every 20 min) for 4 h, and this dataset was treated as a single observation (sample; Appendix S6). Observations were conducted on warm, sunny, windless days. Altogether, 94 observations/samples were recorded from June to August in 2021 and 2022. We collected data from at least 7 samples per selected plant species, and each plot was used only once. Based on this dataset, we calculated the number of butterfly species, number of individuals and Shannon index values for every sample. Mean values (with 95% confidence intervals) of those three indices were calculated for each plant species. Differences in butterfly abundance, species richness and Shannon index between particular species of flowers were tested using the Kruskal‐Wallis test. Post hoc testing was done using Dunn's test in the Statistica 6 package.

## 3 Results

**FIGURE 1:**
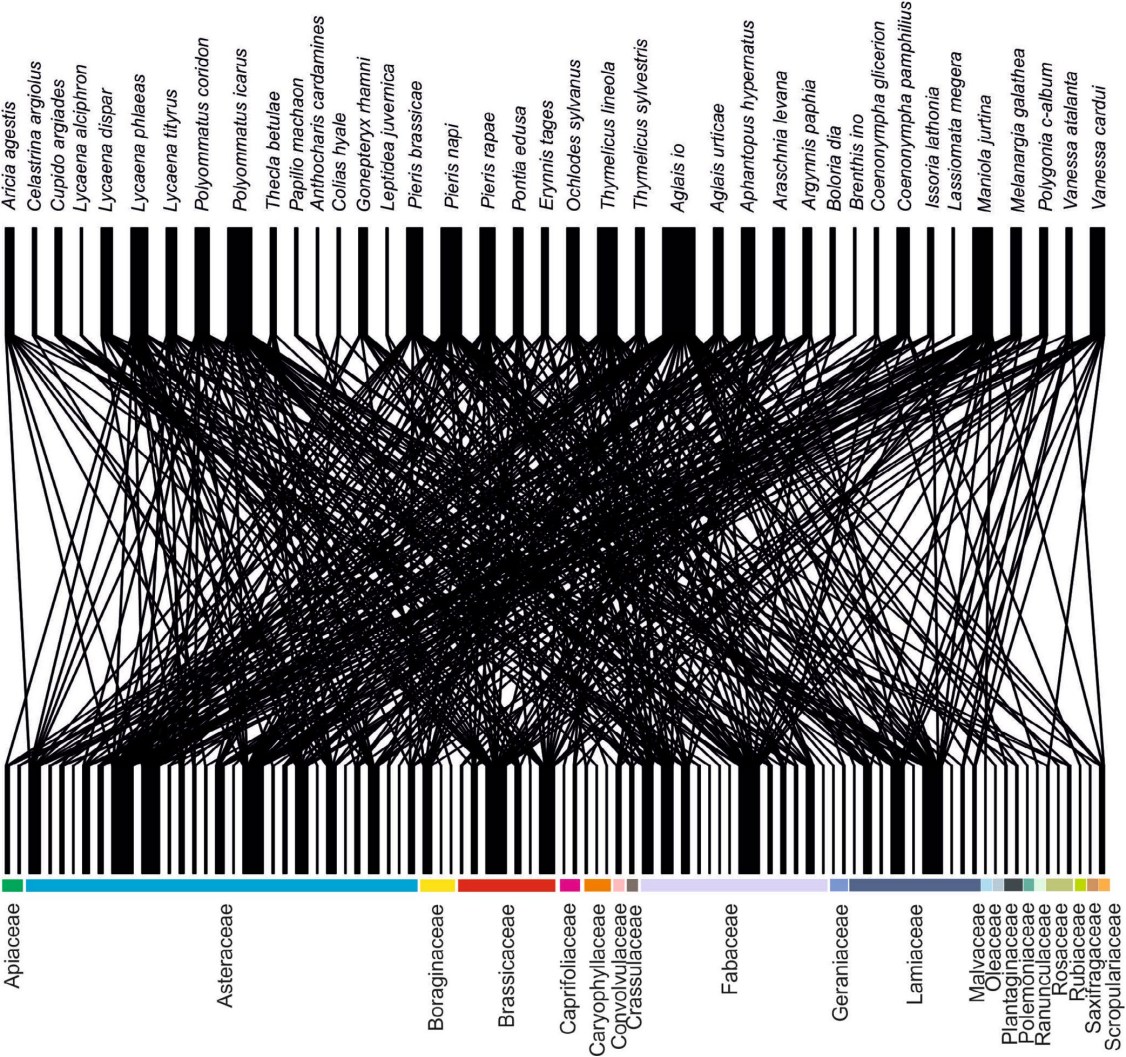
Bipartite plot showing relationships between flowering plants and butterflies. The thickness of the black lines points at the most commonly used plants and butterflies that were utilising the larges number of plant species.

We have recorded flower visits for 39 butterfly species on 81 species of plants, representing 19 families and 16 orders (Appendices [Link], [Link], [Link], [Link]). Six butterfly species: *Melitaea cinxia*, *Nymphalis antiopa* , *Satyrium pruni*, *Saturium w‐album*, *Apatura ilia* and *Pararge aegeria* were never observed on flowers in Łódź. Butterflies mostly visited herbaceous plants (75 species), 5 shrub species and 1 tree species. The highest number of species represented three families, Asteraceae (27 species), Fabaceae (13 species) and Lamiaceae (9 species). Representatives of 2 major plant clades dominated the diets of butterflies in Łódź, namely: asterids (49 species) and rosids (26 species; Figure 1). We noted a group of plants that attracted 15 or more species of butterflies. The largest number of butterfly species was observed on Asteraceae like *Centaurea stoebe* (23 species), *Jasione montana* (22 species), *Cirsium arvense* (19 species), *Achillea vulgaris* (12 species), *Tanacetum vulgare* (11 species) and on flowers of 7 species representing other families, including: Brassicaceae ( *Berteroa incana* —22 species), Fabaceae ( *Trifolium pratense* 22—species), Lamiaceae ( *Origanum vulgare* —21 species, *Lavendula officinalis* 14 species), Fabaceae ( *Lotus corniculatus* —11 species, *Medicago sativa* /*falcata* violet—12 species) and Boraginaceae ( *Echium vulgare* —9 species). In general, these plant species attracted representatives of all butterfly families recorded in Łódź, except *Tanacetum vulgare* which did not attract Pieridae and Hesperidae, as well as *Achillea vulgaris* and *Lavendula officinalis* which did not attract Hesperidae. Plant families that attracted the highest number of butterfly species included Asteraceae (34 species of butterflies), Fabaceae (33 species of butterflies), Brassicaceae (25 species of butterflies) and Lamiaceae (25 species of butterflies).

In general, butterflies were observed on 9 flower colours, including pink (21 species of plants), yellow (20 species of plants), white (17 species of plants), violet (13 species of plants), white/yellow (2 species of plants), orange (2 species of plants), blue (2 species of plants), grey (1 species of plant), red (1 species of plant). Two genera (*Aster* and *Zinnia*) were represented by various colour forms and were not classified into any of the previous categories (Appendices [Link], [Link], [Link]). In general, species visited shallow (47 species) and medium‐depth (32 species) flowers. Only two taxa ( *Saponaria officinalis* and *Phlox*) represented plants with deep flowers (Appendices [Link], [Link], [Link]).

The number of plants visited by particular butterflies varied strongly (Appendix S3). Eighteen species of butterflies were observed on more than 10 species of plants. The butterflies with the highest number of visited plants were *Aglais io* (35 species, 13 families), *Polyommatus icarus* (25 species, 6 families), *Pieris napi* (26 species, 6 families), *Thymelicus lineola* (21 species, 7 families) and *Maniola jurtina* (21 species, 8 families). In contrast, species like *Lycaena alciphron*, *Brenthis ino*, *Lasiommata megera*, *Anthocharis cardamines*, *Leptidea juvernica*, *Celastrina argiolus* , *Papilio machaon* , *Colias hyale*, *Boloria dia* and *Coenonympha glycerion* were recorded on less than 5 species of plants.

**FIGURE 2:**
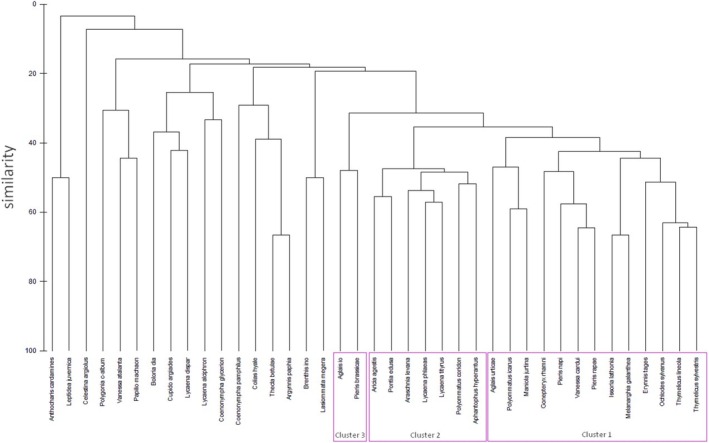
Bray–Curtis similarity of butterflies based on plant species (clusters are marked by frames).

Analysis of similarity demonstrated ecological affinities between butterfly species (Figure 2). Three groups (clusters 1, 2 and 3) of butterflies visiting flowers of several or more species of plants were recorded at 40%–50% similarity. Cluster 1 was associated with species like *Centaurea stoebe* , *Jasione montana* , *Trifolium pratense* and *Origanum vulgare* , but the large majority of species were also visiting flowers of *Cirsium arvense* and *Echium vulgare* . The second cluster group of butterfly species was associated with almost the same group of plants, including *Jasione montana* , *Cirsium arvense* , *Origanum vulgare* and *Centaurea stoebe* , but they were never recorded on *Echium vulgare* and *Trifolium pratense*, and only one of them (*Pontia edusa*) was observed on *Lavendula officinalis*. At the same time, most of these were observed on *Achillea vulgaris*. The third cluster of generalists grouped only two butterflies that differed strongly in the number of visited plants ( *A. io* —35 species, *P. brassicae* —17 species) but both of them were not observed on *Jasione montana* and *Echium vulgare* and were found on ornamental garden plants, namely *Agastache mexicana*, *Phlox* and *Aster*. Six other small clusters grouped various species that were observed on a low number of plants or species that were rarely recorded in the city, and the low number of their observations on flowers is most likely associated with their overall rarity on observed sites (for quantitative data of each butterfly at investigated sites, please see Pietrzak and Pabis (2024)).

**FIGURE 3:**
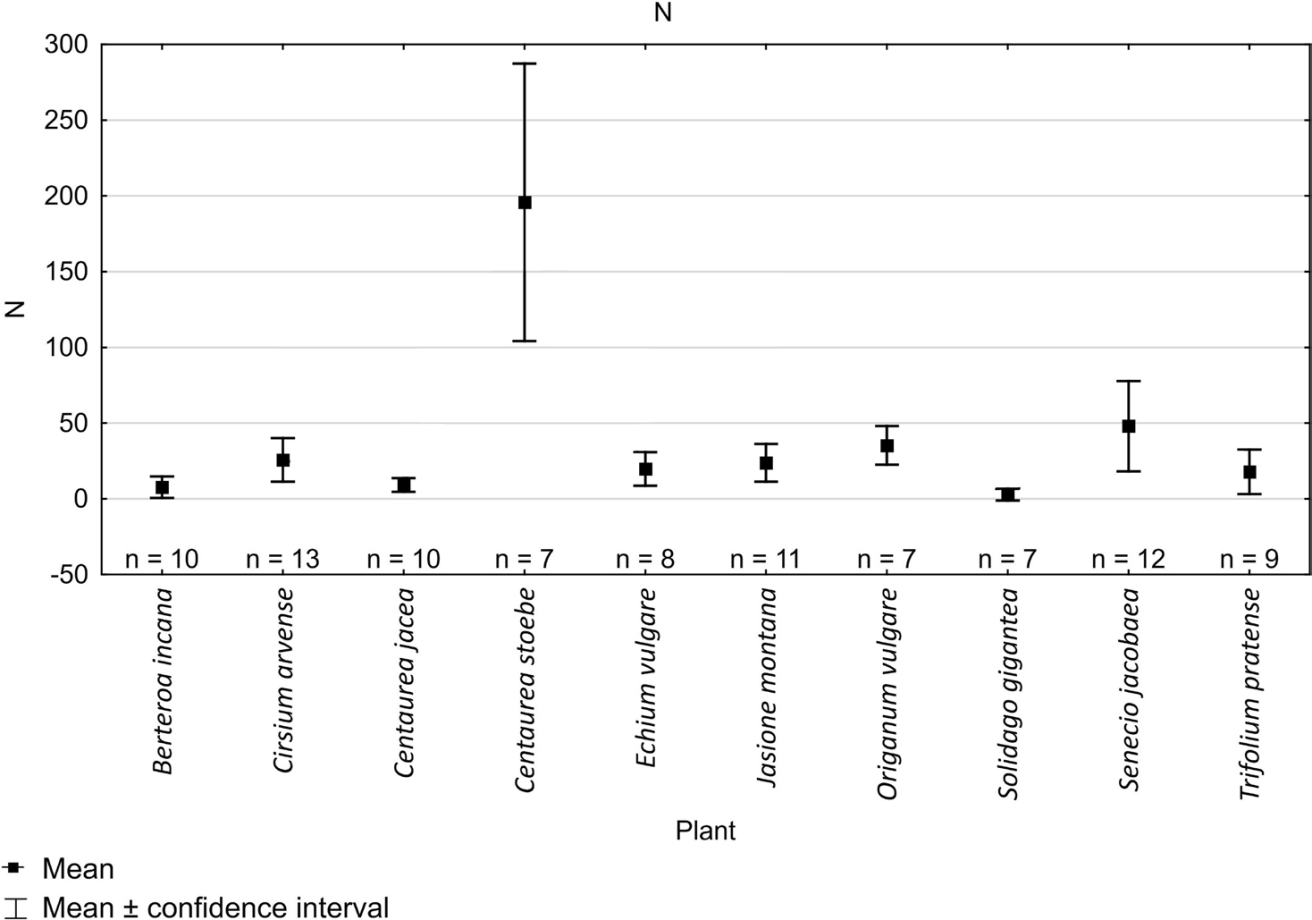
Mean abundance (*N*) of butterflies recorded on different species of plants.

**FIGURE 4:**
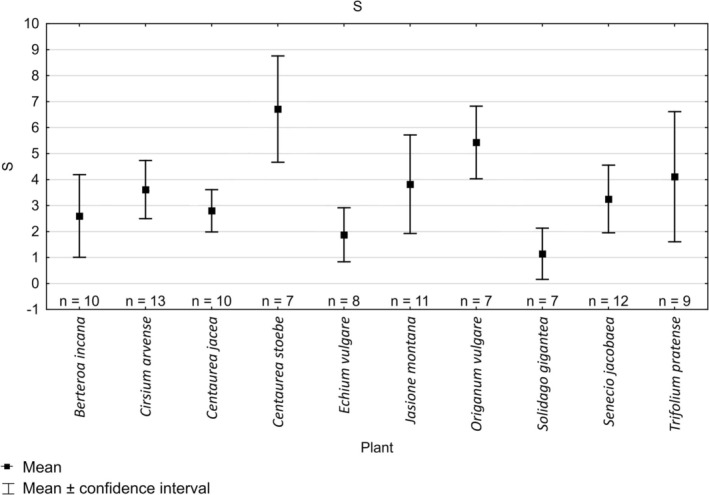
Mean species richness (*S*) of butterflies recorded on different species of plants.

**FIGURE 5:**
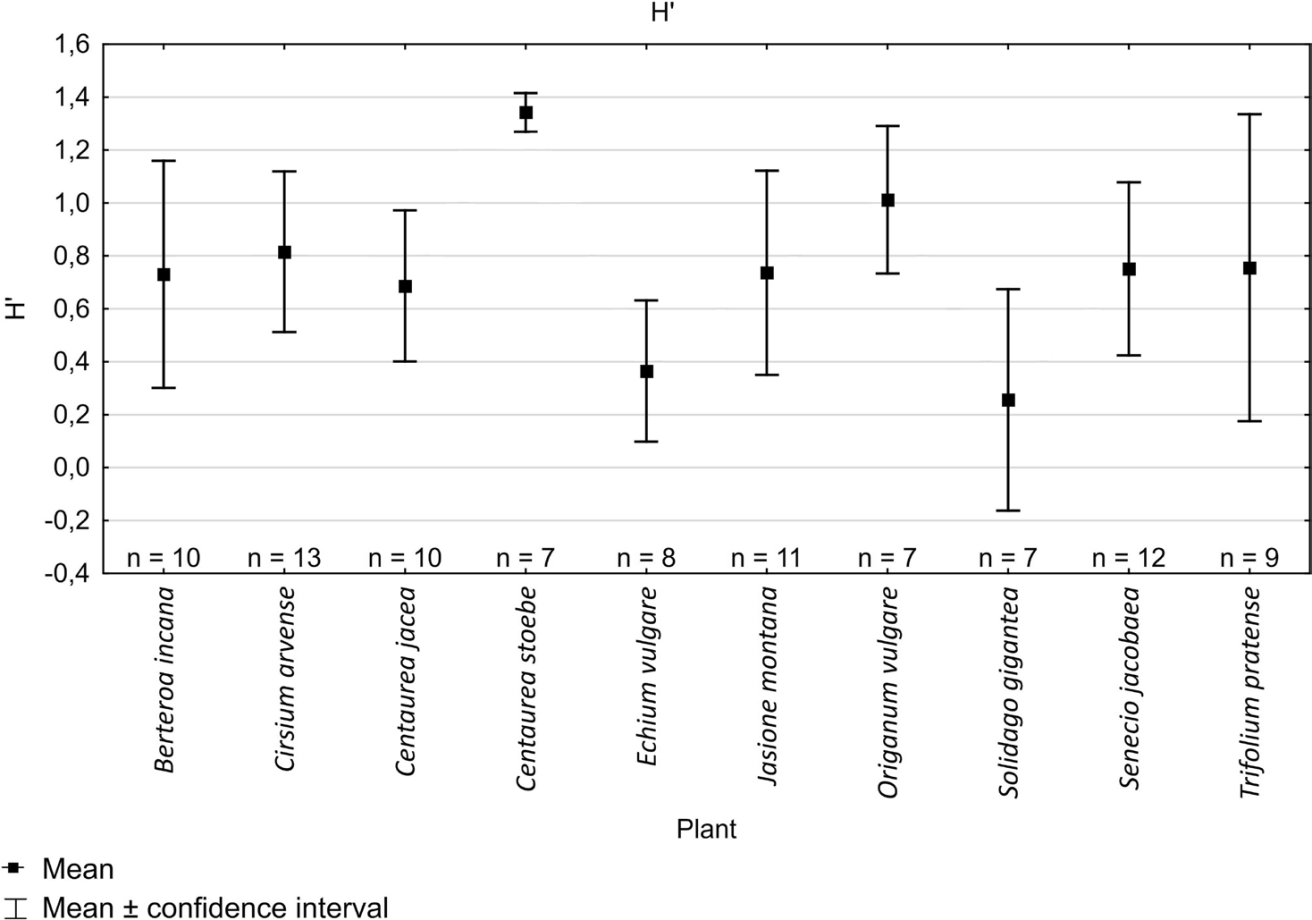
Mean values of Shannon index (*H*) on different species of plants.

Quantitative analysis of flower visits showed that the highest mean number of individuals visiting the plot of plants within the 4‐h period was recorded for *Centaurea stoebe* with 195.9 ± 99.1 ind./4 h (Figure 3). Species richness (*S* = 6.7 ± 2.2) and Shannon index were also the highest (*H*′ = 1.3 ± 0.1; Figures 4 and 5). Altogether, 14 species of butterflies were recorded on this plant (Appendix S6). The plant with the second highest number of observed individuals and high richness and diversity was *Senecio jacobaea* (*N* = 48.0 ± 46.9 ind./4 h *S* = 3.2 ± 2.1 *H* = 0.8 ± 0.5). Values of all three indices recorded for other species of plants were relatively similar (Figures 3, 4, 5). Kruskal‐Wallis test and Dunn's test revealed no significant differences for abundance, species richness or Shannon index values except for *Centaurea stoebe* and *Solidago gigantea* for all indices, *Solidago gigantea* and *Origanum vulgare* for species richness, *Centaurea stoebe* and *Echium vulgare* for number of individuals and Shannon index (Appendix S5).

## 4 Discussion

### 4.1 Richness of Flowering Plants Utilised by Urban Butterflies

Eighteen species of butterflies were observed on more than 10 species of flowers, while 17 species were observed visiting the flowers of five or more plant families (Appendix S3). The wide number of flowering plants used by those butterflies is also linked with their generally high abundance and/or frequency of occurrence in Łódź (Sobczyk et al. 2017) and in the studied wastelands (Pietrzak and Pabis 2024). This observation is generally consistent with the common notion that generalists are better adapted to fragmented and disturbed urban landscapes (Clark et al. 2007; Di Mauro et al. 2007; Callaghan et al. 2021). Interestingly, the majority of species visited plants (Appendices S3 and S4) that are not used by their caterpillars (Sielezniew and Dziekańska 2010; Buszko and Masłowski 2008), which increases their environmental plasticity and the range of habitat used in the adult stage. The majority of species recorded in Łódź also did not display strong flower colour associations, a factor that may limit the distribution of some butterflies (Tiple et al. 2006), although all were associated with shallow and medium depth flowers which can not constitute a limiting factor in Łódź because such species are common in the whole city (Witosławski 2006). Other functional traits and elements of the life cycle are probably crucial for common urban dwellers. This includes mobility, dispersal abilities and availability of caterpillar host plants (Börschig et al. 2013; Kőrösi et al. 2022; Pla‐Narbona et al. 2021). Flower availability may not be the most important factor influencing butterfly distribution in some cities (Bergerot et al. 2010), but was found to be a significant factor in others (Stewart et al. 2018). Nevertheless, the feeding preferences of adults could certainly be an important factor in structuring the diversity and distribution of butterfly communities (Steffan‐Dewenter and Westphal 2008; Thomas et al. 2011; Curtis et al. 2015; Martinez‐Adriano et al. 2018). On the other hand, not all of the most common butterfly species in Łódź (Pietrzak and Pabis 2024) were recorded on a high number of plants. For example, one of the most abundant species, *C. pamphilus* (Pietrzak and Pabis 2024) was observed on 10 species of plants and was not observed on common urban flowers like *Jasione montana*, *Knautia arvensis* , *Cirsium* or *Cantaurea stoebe* or ornamented garden plants like *Lavendula officinalis*, despite the fact that it was already observed feeding on those plants in natural habitats and gardens (Buszko and Masłowski 2008). Other common butterflies like *M. jurtina*, *A. hyperanthus* and *L. phleas* utilised a large number of plants, but these plants mostly represented one family (in this case Asteraceae), which are known as good nectar sources for butterflies (Tudor et al. 2004; Shihan and Kabir 2015; Buszko and Masłowski 2008; Venjakob et al. 2021) and are generally common in Łódź (Witosławski 2006) and other urban areas (Nikolić and Stevović 2015; Dubois and Cheptou 2017; Géron et al. 2021). Their pollen is rarely exploited by generalist bees, probably as a result of chemical defences (Van der Planck et al. 2020), but this nectar is rich in amino acids essential for pollinators and rich in hexoses (Venjakob et al. 2021). In contrast, species like *A. io* or *A. urticae* were observed on many species representing different families and orders of plants despite the fact that those butterflies were less abundant in the investigated wastelands (Pietrzak and Pabis 2024). Therefore, flower preferences were probably not crucial for their distribution because they can actively search for resources over large distances (Bartonova et al. 2014). It is also worth mentioning that species recorded on a small number of flowers are not necessarily flower specialists. Most of them were recorded so rarely in Łódź (Pietrzak and Pabis 2024) that the number of their feeding events is likely strongly biased by the limited number of observations, except for species like *P. aegeria* and *A. ilia*, which rarely visit flowers (Buszko and Masłowski 2008). On the other hand, almost all butterflies recorded in investigated wastelands were abundant and/or commonly observed, and therefore, the above‐mentioned bias concern only a few species, like *Carcharodus alceae*, *Lycaena alciphron*, *Satyrium pruni*, *Satyrium w‐album*, *Thecla betulae*, *Brenthis ino*, *Coenonympha glycerion* and *Melitaea cinxia* (Pietrzak and Pabis 2024).

It is very difficult to compare our results with earlier studies because only one European research paper on flower preferences has been based on long‐term observations and was performed a far distance from Łódź and in a completely different habitat (Tudor et al. 2004). Earlier data from Poland can also not be linked with particular habitats (Sielezniew and Dziekańska 2010; Buszko and Masłowski 2008). None of the previous analyses were done in urban or suburban areas, except for studies performed in the gardens (Di Mauro et al. 2007; Marquardt et al. 2021). Nevertheless, our results substantially increased the knowledge of the diversity of flowers used by almost all recorded butterflies, even when compared with the known diet for Polish flora (Sielezniew and Dziekańska 2010; Buszko and Masłowski 2008). For example, earlier long‐term studies suggested that *A. paphia* is a flower specialist associated with *Rubus* (Tudor et al. 2004). In Poland, it was observed on *Anthriscus sylvestris* , *Centaurea*, *Cirsium*, *Eupatorium cannabinum* or *Knautia arvensis* (Buszko and Masłowski 2008), while in Łódź it was observed on 9 species of plants representing 5 families, including species never mentioned previously as part of its diet, such as *Tanacetum vulgare* , *Allium*, *Solidago canadensis* and *S. gigantea* (Appendix S3). A narrow number of utilised flowers was also recorded previously for *A. hyperanthus*, *G. rhamni*, and even *A. io* in the Wyre Forest nature reserve on the British Isles (Tudor et al. 2004), which might suggest that flower preferences are strongly dependent on the habitat type because *A. io* is generally common on a wide variety of flowers (Buszko and Masłowski 2008).

The largest number of species was recorded on plants representing Asteraceae, some of them like *Centaurea stoebe* were visited by over 20 species of butterflies. Several species of plants attracted more than 10 species of butterflies, and the urban table was generally dominated by Asteraceae, Fabaceae, Brassicaceae, Lamiaceae and Boraginaceae. The list of the most often visited species of flowers differed from observations performed in woodland habitats on the British Isles, where the largest number of flower visits was recorded on *Cirsium*, yellow Asteraceae, *Rubus fruticosus* , *Ajuga reptans* and *Calluna vulgaris* (Tudor et al. 2004). Nevertheless, butterfly–flower associations observed in Łódź might be explained by features of particular plants. Species like *Lotus corniculatus* or *Trifolium pratensis* have very high carbohydrate concentrations and are attractive to pollinating insects; moreover, families like Lamiaceae, Asteraceae and Fabaceae have high carbohydrate‐amino acids ratios of 19:1, 6:1 and 5:1, respectively (Venjakob et al. 2022), which supports our findings that they are the main sources of food for many butterflies. Recent studies conducted in Poznań (western Poland) also showed that plants representing Boraginaceae, Asteraceae and Lamiaceae are important for pollinators (Dylewski et al. 2020), although the authors did not analyse specific preferences of butterfly species. Brassicaceae are an important food source for many pollinators (mostly bees and hoverflies) but rarely for butterflies (Badenes‐Pérez 2022), although their presence in the diet of European butterflies is not surprising (Buszko and Masłowski 2008).

Wasteland vegetation acts as flower resources in the management of urban green spaces. Urban garden plants were often mentioned among the main flower resources for butterflies in cities (Di Mauro et al. 2007; Marquardt et al. 2021), but those species are often planted in the strict city centres, botanical gardens or larger parks. These areas are not necessarily a perfect habitat for butterflies. It is mostly due to intensive management practices, regular grass mowing and lack of caterpillar host plants (Öckinger et al. 2009; Aguilera et al. 2019). Similar observations were conducted in Łódź, where butterfly fauna of large parks was less speciose (Sobczyk et al. 2017) than faunas associated with small wastelands (Pietrzak and Pabis 2024). Our study demonstrated that such habitats are becoming a blooming urban table that can attract various species of butterflies to plants that are often neglected in the management of urban areas. Even if the municipal authorities are trying to maintain the biodiversity of insects within the city borders to enhance the well‐being of the citizens according to the latest recommendations, the wasteland floras are rarely taken into account (Bellamy et al. 2017; Samways et al. 2020). Therefore, our results might change the common practices surrounding the management of urban green spaces, pointing at possible new directions and more extensive protection of wastelands or ruderal sites as they act as important resources for pollinators. Moreover, recent studies demonstrated that many garden plants which are found in garden centres are unattractive to flower‐visiting insects (Garbuzov et al. 2017). Some other studies suggested that urban green spaces are hot spots of floral resource diversity but not hot spots for the quantity of nectar supplies (Tew et al. 2021). In contrast, some of the horticulturally modified plants are more or equally attractive to insects (Garbuzov and Ratnieks 2014; Marquardt et al. 2021). Nevertheless, our results showed that we do not need ornamental plants to attract butterflies into cities. Moreover, we already know that preferences for exotic flowers do not promote the urban affinity of European butterflies (Bergerot et al. 2010). Our results should also be viewed in the light of planning urban meadows or urban green spaces in general, while seed mixes often contain seeds of *T. pratense* , *L. corniculatus* or *Centaurea* that received many visits by butterflies in Łódź, but can also contain various alien species which should be avoided (Hicks et al. 2016). Communities of native ruderal plants might constitute a good alternative or supplement to seed mixes. Therefore, we postulate that a greater selection of plants in urban green spaces and the use of species that are already present in the cities, can attract many species of butterflies, but at the same time might be attractive to humans, for example: *Jasione montana* , *Knautia arvensis* or *Berteroa incana* .

### 4.2 Flower Colour Associations on Urban Wastelands

Studies from India and Nepal demonstrated that butterflies might prefer blue, yellow, red and violet flowers over white and pink flowers (Tiple et al. 2006; Subedi et al. 2021), while studies from Turkey demonstrated a preference for yellow and pink flowers and avoidance of red flowers (Yurtsever et al. 2010). We did not record such patterns in Łódź, which is congruent with growing evidence that tight colour‐based plant–pollinator associations are generally rare because most pollinators are flower generalists (Reverté et al. 2016); therefore, flower visits might also depend on local plant availability or general diversity of flower resources (Sexton et al. 2025), although flowers of different colours were recorded on all investigated wastelands. Therefore, flower colour preferences, even when observed, are not accompanied by constancy (Pohl et al. 2011). Only a few of the most common (and therefore most regularly observed on flowers during the study period) butterflies observed in Łódź (Pietrzak and Pabis 2024) demonstrated narrower associations with flower colour. For example, *G. rhamni* and *M. galathea* were found on violet and pink flowers, while the majority of other species were observed on flowers of many different colours. Therefore, other factors, like above‐discussed nectar reward, were probably more important for flower–butterfly associations.

### 4.3 Quantitative Analysis of Flower Visits

Interesting questions arose during our attempt at quantitative analysis of flower visits. In contrast to studies carried out in gardens or on experimentally prepared research plots (Shackleton and Ratnieks 2016; Wijesinghe et al. 2020; Marquardt et al. 2021), where observers can indirectly control the number of flowers and/or the size of plots during plant observations, we were bound to the irregularity of plots and their random (or uniform) distribution. This is a key inconvenience that needs to be addressed when attempting to perform a methodologically consistent study. However, some of these problems were already noted during observations performed on garden plants, and it is generally difficult to propose fully reliable field methods for measuring plant attractiveness to pollinators (Wijesinghe et al. 2020; Erickson, Grozinger, and Patch 2022; Erickson, Junker, et al. 2022). For example, in the case of some particular plant species (e.g., *Solidago*, *Centaurea stoebe* or *Berteroa incana* ), locating a single, well‐separated plot can be quite difficult because these plants densely cover large areas. We have also observed disproportions between the total number of butterfly species observed on particular species of plants during long‐term qualitative observations and the number of species observed during quantitative studies conducted in the restricted time period. For instance, our qualitative results demonstrated that *Centaurea stoebe* was generally visited by 23 species of butterflies, *Berteroa incana* by 22 species and *Solidago* by 10 species, while only 14, 11 and 5 species, respectively, were documented during observations of selected plots during the quantitative research period. Such differences may result from the composition of local species pools or may illustrate problems in observations of naturally less abundant species (Haddad et al. 2008). Therefore, these two datasets should be viewed together to provide a comprehensive picture.

The problem of relationships between the composition of pollinator assemblages and flower integration might also be important (Ordano et al. 2008; González et al. 2015). Adopting a uniform and comparable unit that reflects a plot's attractiveness measured in the number of resources (e.g., number of flowers on a given surface) is almost impossible. For example, the inflorescence of *Trifolium pratense* consists of a small head composed of tubular flowers, while the inflorescence of *Solidago* is huge and covered with hundreds of flowers. Therefore, conclusions drawn from the comparisons of different plants should be treated with particular caution. However, it is worth mentioning that the large inflorescence of *Solidago*, or inflorescence of *Achillea millefolium* , attracted only 5 and 4 species, respectively, while plants with small inflorescences like *Centaurea stoebe* , *Cirsium arvense* and *Senecio jacobea* attracted not only a higher number of species but also considerably higher numbers of individuals.

Despite these problems, such a quantitative approach seems to be highly desirable and allows researchers to select species of plants that can be used in the management of urban green spaces. Nevertheless, we certainly need to field test various methodological approaches.

## 5 Concluding Remarks

Despite the fact that the first part of our study was based on qualitative observations, it still represents an exceptional dataset for European urban butterfly communities. The majority of earlier data about the flower associations of European butterflies are not based on regular monitoring of particular habitats but rather on occasional observations, most probably conducted in natural habitats (e.g., Sielezniew and Dziekańska 2010; Buszko and Masłowski 2008). Moreover, such data are generally rare, highly scattered, and often neglected, even in identification field guides for European butterflies (e.g., Tolman 1997; Lafranchis 2004). As a result, the knowledge about flower preferences is not accessible to the wider group of butterfly watchers, is rarely catalogued even by specialists, and cannot be used in conservation actions, especially in altered ecosystems like large cities. Data on flower associations are also an important source of information for studies of functional diversity, which is becoming a very important trend in current entomological studies (Aguirre‐Gutiérrez et al. 2017). In urban habitats, such data constitute important information for conservation planning, management of green spaces and ecosystem services (Paudel and States 2023). Furthermore, it is very important to link this knowledge with the management of urban green spaces and the planning of conservation strategies not only in urban areas but also in natural ecosystems.

## Author Contributions

**Sylwia Pietrzak:** conceptualization (equal), formal analysis (equal), investigation (lead), methodology (equal), writing – original draft (equal), writing – review and editing (equal). **Krzysztof Pabis:** conceptualization (equal), formal analysis (equal), methodology (equal), supervision (lead), writing – original draft (equal), writing – review and editing (equal).

## Conflicts of Interest

The authors declare no conflicts of interest.

## Supporting information


**Appendix S1:** ece372088‐sup‐0001‐AppendixS1.docx.

**Appendix S2:** ece372088‐sup‐0001‐AppendixS2.docx.

**Appendix S3:** ece372088‐sup‐0001‐AppendixS3.docx.

**Appendix S4:** ece372088‐sup‐0001‐AppendixS4.docx.

**Appendix S5:** ece372088‐sup‐0001‐AppendixS5.docx.

**Appendix S6:** ece372088‐sup‐0001‐AppendixS6.docx.

## Data Availability

All raw data used in this study are available in the Appendices S1, S2 and S6 of this article.
