# Supplementary material for: Blooming Urban Table: Flower Resources for Butterflies in Small Wastelands of a Large European City
Source: Ecol Evol. 2025 Sep 15;15(9):e72088. doi: 10.1002/ece3.72088 (PMC12434404; doi:10.1002/ece3.72088)
Supplement: Supplementary file 3 — Appendix S3: ece372088‐sup‐0001‐AppendixS3.docx. [file ECE3-15-e72088-s003.docx]

Appendix 3. Flower-butterfly associations recorded in Łódź.

|  | | | | | | | | |
| --- | --- | --- | --- | --- | --- | --- | --- | --- |
| **Species** | **No. of plant species** | **Plant families** | **No. of plant families** | **No. of plant orders** | **Plant orders** | **No. of plant colors** | **Flower color** | **Flower depth** |
| *Erynnis tages* | 7 | Asteraceae (2 species), Boraginaceae (2 species), Fabaceae (2 species), Lamiaceae (1 spec ies) | 4 | 4 | Asterales (2 species)  Boraginales (2 species)  Fabales (1 species)  Lamiales (1 species) | 4 | blue (1 species)  pink (2 species)  violet (2 species)  yellow (2 species) | shallow (3 species)  medium (4 species) |
| *Ochlodes sylvanus* | 13 | Asteraceae (3 species), Boraginaceae (1 species), Fabaceae (1 species), Lamiaceae (1 species)  Rosaceae (1 species) | 5 | 5 | Asterales (3 species)  Boraginales (1 species)  Fabales (1 species)  Lamiales (1 species)  Rosales (1 species) | 4 | pink (5 species)  violet (5 species)  white (1 species)  yellow (2 species) | medium (9 species)  shallow (4 species) |
| *Thymelicus lineola* | 21 | Asteraceae (6species)  Boraginaceae (2 species)  Brassicaceae (1 species)  Caryophyllaceae (1 species)  Fabaceae (7 species)  Lamiaceae (2 species)  Plantaginaceae (2 species) | 7 | 6 | Asterales (6 species)  Boraginales (2 species)  Brassicales (1 species)  Caryophyllales (1 species)  Fabales (7 species)  Lamiales (4 species) | 5 | blue (1 species)  pink (7 species)  violet (7 species)  white (2 species)  yellow (4 species) | medium (13 species)  shallow (8 species) |
| *Thymelicus sylvestris* | 9 | Asteraceae (4 species)  Boraginaceae (1 species)  Caryophyllaceae (1 species)  Fabaceae (3 species) | 4 | 4 | Asterales (4 species)  Boraginales (1 species)  Caryophyllales (1 species)  Fabales (3 species) | 3 | pink (4 species)  violet (4 species)  yellow (1 species) | medium (5 species)  shallow (4 species) |
| *Papilio machaon* | 3 | Fabaceae (1 species)  Lamiaceae (2 species) | 2 | 2 | Fabales (1 species)  Lamiales (2 species) | 2 | pink (2 species)  violet (1 species) | medium (3 species) |
| *Anthocharis cardamines* | 2 | Brassicaceae (2 species) | 1 | 1 | Brassicales (2 species) | 1 | white (2 species) | shallow (2 species) |
| *Colias hyale* | 3 | Asteraceae (2 species)  Fabaceae (1 species) | 2 | 2 | Asterales (2 species)  Fabales (1 species) | 2 | pink (1 species)  yellow (2 species) | medium (1 species)  shallow (2 species) |
| *Gonepteryx rhamni* | 9 | Asteraceae (2 species)  Boraginaceae (1 species)  Fabaceae (4 species)  Lamiaceae (2 species) | 4 | 4 | Asterales (2 species)  Boraginales (1 species)  Fabales (4 species)  Lamiales (2 species) | 2 | pink (3 species)  violet (6 species) | medium(7 species)  shallow (2 species) |
| *Leptidea juvernica* | 2 | Brassicaceae (1 species)  Fabaceae (1 species) | 2 | 2 | Brassicales (1 species)  Fabales (1 species) | 2 | white (1 species)  yellow (1 species) | medium (1 species)  shallow (1 species) |
| *Pieris brassicae* | 17 | Asteraceae (6 species)  Brassicaceae (1 species)  Boraginaceae (1 species)  Fabaceae (3 species)  Lamiaceae (4 species)  Polemoniaceae (1 species)  Scrophulariaceae (1 species) | 7 | 6 | Asterales (6 species)  Brassicales (1 species)  Boraginales (1 species)  Fabales (3 species)  Lamiales (5 species)  Ericales (1 species) | 6 | blue (1 species)  pink (6 species)  various (1 species)  violet (5 species)  white (2 species)  yellow (1 species) | Deep (1 species)  Medium (9 species)  Shallow (5 species) |
| *Pieris napi* | 21 | Asteraceae (7 species)  Brassicaceae (2 species)  Convolvulaceae (1 species)  Fabaceae (4 species)  Geraniaceae (2 species)  Lamiaceae(5 species) | 6 | 6 | Asterales (7 species)  Brassicales (2 species)  Solanales (1 species)  Fabales (4 species)  Geraniales (2 species)  Lamiales (5 species) | 5 | orange (1 species)  pink (8 species)  violet (6 species)  white (3 species)  yellow (3 species) | medium (11 species)  shallow (10 species) |
| *Pieris rapae* | 16 | Asteraceae (6 species)  Boraginaceae (1 species)  Brassicaceae (3 species)  Fabaceae (3 species)  Lamiaceae (3 species) | 5 | 5 | Asterales (6 species)  Boraginales (1 species)  Brassicales (3 species)  Fabales (3 species)  Lamiales (3 species) | 4 | pink (4 species)  violet (6 species)  white (1 species)  yellow (3 species) | medium (7 species)  shallow (8 species) |
| *Pontia edusa* | 10 | Asteraceae (5 species)  Brassicaceae (2 species)  Caryophyllaceae (1 species)  Fabaceae (1 species)  Lamiaceae (1 species) | 5 | 5 | Asterales (5 species)  Brassicales (2 species)  Caryophyllales (1 species)  Fabales (1 species)  Lamiales (1 species) | 4 | pink (2 species)  violet (4 species)  white (3 species)  yellow (1 species) | deep (1 species)  medium (2 species)  shallow (7 species) |
| *Aricia agestis* | 9 | Asteraceae (6 species)  Brassicaceae (1 species)  Fabaceae (1 species)  Lamiaceae (1 species) | 4 | 4 | Asterales (6 species)  Brassicales (1 species)  Fabales (1 species)  Lamiales (1 species) | 4 | pink (2 species)  violet (2 species)  white (2 species)  yellow (3 species) | medium (2 species)  shallow (7 species) |
| *Celestina argiolus* | 4 | Asteraceae (1 species)  Fabaceae (2 species)  Lamiaceae (1 species) | 3 | 3 | Asterales (1 species)  Fabales (2 species)  Lamiales (1 species) | 3 | pink (1 species)  white (1 species)  yellow (2 species) | medium (3 species)  shallow (1 species) |
| *Cupido argiades* | 7 | Brassicaceae (1 species)  Fabaceae (6 species) | 2 | 2 | Brassicales (1 species)  Fabales (6 species) | 4 | pink (2 species)  violet (1 species)  white (2 species)  yellow (2 species) | medium (6 species)  shallow (1 species) |
| *Lycaena alciphron* | 2 | Asteraceae (1 species)  Brassicaceae (1 species) | 2 | 2 | Asterales (1 species)  Brassicales (1 species) | 2 | pink (1 species)  white (1 species) | shallow (2 species) |
| *Lycaena dispar* | 12 | Asteraceae (3 species)  Brassicaceae (3 species)  Fabaceae (5 species)  Rosaceae (1 species) | 4 | 4 | Asterales (3 species)  Brassicales (3 species)  Fabales (5 species)  Rosales (1 species) | 5 | pink (2 species)  violet (1 species)  white (4 species)  white+yellow (1 species)  yellow (4 species) | medium (5 species)  shallow (7 species) |
| *Lycaena phlaeas* | 17 | Apiaceae (1 species)  Asteraceae (11 species)  Brassicaceae (2 species)  Fabaceae (2 species)  Lamiaceae (1 species) | 5 | 5 | Apiales (1 species)  Asterales (11 species)  Brassicales (2 species)  Fabales (2 species)  Lamiales (1 species) | 5 | pink (3 species)  violet (3 species)  white (5 species)  white+yellow (1 species)  yellow (5 species) | medium (3 species)  shallow (14 species) |
| *Lycaena tityrus* | 11 | Asteraceae (6 species)  Brassicaceae (1 species)  Caryophyllaceae (1 species)  Fabaceae (1 species)  Lamiaceae (1 species)  Rosaceae (1 species) | 6 | 6 | Asterales (6 species)  Brassicales (1 species)  Caryophyllales (1 species)  Fabales (1 species)  Lamiales (1 species)  Rosales (1 species) | 5 | pink (1 species)  violet (3 species)  white (4 species)  white+yellow (1 species)  yellow (2 species) | medium (2 species)  shallow (9 species) |
| *Polyommatus coridon* | 15 | Asteraceae (9 species)  Boraginaceae (1 species)  Brassicaceae (1 species)  Fabaceae (2 species)  Lamiaceae (1 species)  Rosaceae (1 species) | 6 | 6 | Asterales (9 species)  Boraginales (1 species)  Brassicales (1 species)  Fabales (2 species)  Lamiales (1 species)  Rosales (1 species) | 6 | blue (1 species)  pink (5 species)  violet (2 species)  white (3 species)  white+yellow (1 species)  yellow (3 species) | medium (4 species)  shallow (11 species) |
| *Polyommatus icarus* | 26 | Asteraceae (8 species)  Boraginaceae (2 species)  Brassicaceae (2 species)  Crassulaceae (1 species)  Fabaceae (9 species)  Lamiaceae (4 species) | 6 | 6 | Asterales (8 species)  Boraginales (2 species)  Brassicales (2 species)  Saxifragales (1 species)  Fabales (9 species)  Lamiales (4 species) | 6 | grey (1 species)  orange (1 species)  pink (6 species)  violet (9 species)  white (5 species)  yellow (4 species) | medium (16 species)  shallow (10 species) |
| *Thecla betulae* | 6 | Asteraceae (5 species)  Fabaceae (1 species) | 2 | 2 | Asterales (5 species)  Fabales (1 species) | 4 | pink (1 species)  violet (1 species)  white+yellow (1 species)  yellow (3 species) | medium (1 species)  shallow (5 species) |
| *Aglais io* | 35 | Apiaceae (1 species)  Asteraceae (17 species)  Brassicaceae (1 species)  Caprifoliaceae (1 species)  Crassulaceae (1 species)  Fabaceae (2 species)  Lamiaceae (6 species)  Malvaceae (1 species)  Polemoniaceae (1 species)  Ranunculaceae (1 species)  Rosaceae (1 species)  Saxifragaceae (1 species)  Scrophulariaceae (1 species) | 13 | 11 | Apiales (1 species)  Asterales (17 species)  Brassicales (1 species)  Dipsacales (1 species)  Saxifragales (2 species)  Fabales (2 species)  Lamiales (7 species)  Malvales (1 species)  Ericales (1 species)  Ranunculales (1 species)  Rosales (1 species) | 7 | orange (2 species)  pink (13 species)  red (1 species)  various (2 species)  violet (7 species)  white (4 species)  yellow (6 species) | deep (1 species)  medium (11 species)  shallow (23 species) |
| *Aglais urticae* | 10 | Asteraceae (3 species)  Boraginaceae (1 species)  Brassicaceae (1 species)  Crassulaceae (1 species)  Fabaceae (1 species)  Lamiaceae (2 species)  Scrophulariaceae (1 species) | 7 | 6 | Asterales (1 species)  Boraginales (1 species)  Brassicales (1 species)  Saxifragales (1 species)  Fabales (1 species)  Lamiales (3 species) | 4 | orange (1 species)  pink (4 species)  violet (4 species)  white (1 species) | medium (6 species)  shallow (4 species) |
| *Aphantophus hyperantus* | 14 | Asteraceae (9 species)  Brassicaceae (1 species)  Fabaceae (3 species)  Lamiaceae (1 species) | 4 | 4 | Asterales (9 species)  Brassicales (1 species)  Fabales (3 species)  Lamiales (1 species) | 5 | pink (5 species)  violet (3 species)  white (1 species)  white+yellow (1 species)  yellow (4 species) | medium (4 species)  shallow (10 species) |
| *Araschnia levana* | 12 | Apiaceae (2 species)  Asteraceae (6 species)  Brassicaceae (1 species)  Caryophyllaceae (1 species)  Crassulaceae (1 species)  Lamiaceae (1 species) | 5 | 5 | Apiales (2 species)  Asterales (6 species)  Brassicales (1 species)  Caryophyllales (1 species)  Saxifragales (1 species)  Lamiales (1 species) | 4 | pink (3 species)  violet (2 species)  white (4 species)  yellow (3 species) | deep (1 species)  medium (2 species)  shallow (9 species) |
| *Argynnis paphia* | 9 | Apiaceae (1 species)  Asteraceae (5 species)  Brassicaceae (1 species)  Fabaceae (1 species)  Lamiaceae (1 species) | 5 | 5 | Apiales (1 species)  Asterales (5 species)  Brassicales (1 species)  Fabales (1 species)  Lamiales (1 species) | 3 | pink (2 species)  violet (3 species)  yellow (4 species) | medium (2 species)  shallow (7 species) |
| *Boloria dia* | 4 | Asteraceae (1 species)  Brassicaceae (2 species)  Fabaceae (1 species) | 3 | 3 | Asterales (1 species)  Brassicales (2 species)  Fabales (1 species) | 2 | white (1 species)  yellow (3 species) | medium (1 species)  shallow (3 species) |
| *Brenthis ino* | 2 | Asteraceae (2 species) | 1 | 1 | Asterales (2 species) | 2 | pink (1 species)  violet (1 species) | shallow (2 species) |
| *Coenonympha glycerion* | 4 | Asteraceae (1 species)  Brassicaceae (1 species)  Fabaceae (1 species)  Plantaginaceae (1 species) | 4 | 4 | Asterales (1 species)  Brassicales (1 species)  Fabales (1 species)  Lamiales (1 species) | 2 | violet (2 species)  white (2 species) | medium (1 species)  shallow (3 species) |
| *Coenonympha pamphilus* | 13 | Apiaceae (1 species)  Asteraceae (5 species)  Boraginaceae (1 species)  Brassicaceae (2 species)  Fabaceae (1 species)  Rosaceae (2 species)  Rubiaceae (1 species) | 5 | 5 | Apiales (1 species)  Asterales (5 species)  Boraginales (1 species)  Brassicales (2 species)  Fabales (1 species)  Rosales (2 species)  Gentianales (1 species) | 5 | blue (1 species)  pink (2 species)  violet (1 species)  white (4 species)  white+yellow (1 species)  yellow (4 species) | medium (2 species)  shallow (10 species) |
| *Issoria lathonia* | 6 | Apiaceae (1 species)  Asteraceae (3 species)  Fabaceae (1 species)  Lamiaceae (1 species) | 4 | 4 | Apiales (1 species)  Asterales (3 species)  Fabales (1 species)  Lamiales (1 species) | 3 | pink (4 species)  violet (1 species)  white (1 species) | medium (2 species)  shallow (4 species) |
| *Lasiommata megera* | 2 | Asteraceae (2 species) | 1 | 1 | Asterales (2 species) | 2 | pink (1 species)  yellow (1 species) | shallow (2 species) |
| *Maniola jurtina* | 21 | Asteraceae (11 species)  Brassicaceae (1 species)  Caryophyllaceae (1 species)  Crassulaceae (1 species)  Fabaceae (2 species)  Lamiaceae (3 species)  Oleaceae (1 species)  Scrophulariaceae (1 species) | 8 | 6 | Asterales (11species)  Brassicales (1 species)  Caryophyllales (1 species)  Saxifragales (1 species)  Fabales (2 species)  Lamiales (5 species) | 5 | orange (1 species)  pink (9 species)  violet (5 species)  white (3 species)  yellow (3 species) | medium (8 species)  shallow (13 species) |
| *Melanarghia galathea* | 11 | Asteraceae (9 species)  Fabaceae (1 species)  Lamiaceae (1 species) | 3 | 3 | Asterales (9 species)  Fabales (1 species)  Lamiales (1 species) | 4 | pink (6 species)  violet (2 species)  white (1 species)  yellow (2 species) | medium (2 species)  shallow (9 species) |
| *Polygonia c-album* | 8 | Asteraceae (4 species)  Brassicaceae (1 species)  Lamiaceae (3 species) | 3 | 3 | Asterales (4 species)  Brassicales (1 species)  Lamiales (3 species) | 5 | orange (1 species)  pink (3 species)  various (1 species)  violet (2 species)  white (1 species) | medium (3 species)  shallow (5 species) |
| *Vanessa atalanta* | 6 | Asteraceae (1 species)  Fabaceae (1 species)  Lamiaceae (2 species)  Malvaceae (1 species)  Scrophulariaceae (1 species) | 5 |  | Asterales (1 species)  Fabales (1 species)  Lamiales (3 species)  Malvales (1 species) | 4 | pink (2 species)  various (1 species)  violet (2 species)  yellow (1 species) | medium (4 species)  shallow (2 species) |
| *Vanessa cardui* | 16 | Asteraceae (7 species)  Boraginaceae (2 species)  Brassicaceae (2 species)  Fabaceae (1 species)  Lamiaceae (2 species)  Malvaceae (1 species)  Rosaceae (1 species) | 7 |  | Asterales (7 species)  Boraginales (2 species)  Brassicales (2species)  Fabales (1 species)  Lamiales (2 species)  Malvales (1 species)  Rosales (1 species) | 5 | blue (1 species)  pink (5 species)  violet (4 species)  white (3 species)  yellow (3 species) | medium (5 species)  shallow (11 species) |
