# Supplementary material for: Blooming Urban Table: Flower Resources for Butterflies in Small Wastelands of a Large European City
Source: Ecol Evol. 2025 Sep 15;15(9):e72088. doi: 10.1002/ece3.72088 (PMC12434404; doi:10.1002/ece3.72088)
Supplement: Supplementary file 4 — Appendix S4: ece372088‐sup‐0001‐AppendixS4.docx. [file ECE3-15-e72088-s006.docx]

Appendix 4. Flower associations of butterfly families recorded in Łódź

| **Family** | **No. of plant species** | **No. of plant families** | **Major plant families** | **No. of butterfly species** | **No. of plant species in dominant color groups** | **No. of butterfly species on particular flower color** | **No. of plant species with particular depth of flowers** | **No. of butterfly species recorded on particular depth of flower** |
| --- | --- | --- | --- | --- | --- | --- | --- | --- |
| Hesperidae | 23 | 7 | Fabaceae (8 species),  Asteraceae (6  species) | 4 (all 4 recorded on *Centaurea stoebe*, *Jasione montana*, *Echium vulgare*, *Lotus corniculatus*) | Pink (8 species), Violet (6 species), Yellow (3 species), white (3 species) | Violet (4 species), Yellow (4 species), pink (4 species), white (2 species) | Shallow (8 species), medium (14 species), | Shallow (4 species), medium (4 species), |
| Pieridae | 39 | 9 | Asteraceae (11 species), Fabaceae (8 species), Lamiaceae (6 species), Brassicaceae (5 species) | 8 (5 species recorded on *Cirsium arvense*, *Trifolium pratense* and *Lavendula officinalis*) | Pink (12 species), Violet (8 species), Yellow (7 species), white (7 species) | Yellow (6 species), white (6 species), pink (6 species), violet (5 species) | medium (19 species), shallow (17 species), deep (2 species) | Shallow (8 species), medium (7 species), deep (2 species) |
| Papilionidae | 3 | 2 | Lamiaceae (2 species), Fabaceae (1 species) | 1 (*P. machaon* was recorded only on *Trifolium pratense*, *Lamium purpureum* and *Lavendula officinalis*) | Pink (2 species), violet (1 species) | Pink/violet (1 species) | Medium (3 species) | Medium (1 species) |
| Lycaenidae | 43 | 11 | Asteraceae (14species), Fabaceae (12 species), Caprifoliaceae (5 species) | 10 (8 species recorded on *Berteroa incana*, 6 species on *Tanacetum vulgare*, 5 species on *Achillea vulgaris*, *Jasione montana, Knautia arvensis, Kolkwitzia amabilis, Origanum vulgare*) | Yellow (11 species), white (9 species), violet (9 species), pink (8 species) | Pink (10 species), white (10 species), yellow (9 species), violet (8 species), | Shallow (22 species), medium (21 species), | Shallow (9 species), medium (9 species), |
| Nymphalidae | 62 | 17 | Asteraceae (26 species) Lamiaceae (7 species), Fabaceae (6 species) | 18 (9 species recorded on *Centaurea stoebe, Jasione montana, Berteroa incana, Origanum vulgare*, 8 species found on *Trifolium pratense*, 7 on *Cirsium arvense* and *Lavendula officinalis)* | Pink (17 species), Yellow (15 species), white (11 species), violet (10 species) | Pink (14 species), Violet (14 species), Yellow (11 species), white (11 species), | Shallow (41 species), medium (19 species), deep (2 species) | Shallow (16 species), medium (14 species), deep (2 species) |
