# Supplementary material for: Blooming Urban Table: Flower Resources for Butterflies in Small Wastelands of a Large European City
Source: Ecol Evol. 2025 Sep 15;15(9):e72088. doi: 10.1002/ece3.72088 (PMC12434404; doi:10.1002/ece3.72088)
Supplement: Supplementary file 5 — Appendix S5: ece372088‐sup‐0001‐AppendixS5.docx. [file ECE3-15-e72088-s001.docx]

Appendix 5. Results of post hoc Dunn’s test for species richness (S), Shannon index (H) and number of individuals (N). Statistically significant results are marked in bold red.

|  | | | | | | | | | | |
| --- | --- | --- | --- | --- | --- | --- | --- | --- | --- | --- |
|  | | | | | | | | | | |
| **S (p)** | ***Cirsium arvense*** | ***Echium vulgare*** | ***Senecio jacobaea*** | ***Trifolium pratense*** | ***Jasione montana*** | ***Centaurea jacea*** | ***Berteroa incana*** | ***Centaurea stoebe*** | ***Origanum vulgare*** | ***Solidago gigantea*** |
| ***Cirsium arvense*** |  | 1.00 | 1.00 | 1.00 | 1.00 | 1.00 | 1.00 | 0.98 | 1.00 | 0.79 |
| ***Echium vulgare*** | 1.00 |  | 1.00 | 1.00 | 1.00 | 1.00 | 1.00 | 0.02 | 0.14 | 1.00 |
| ***Senecio jacobaea*** | 1.00 | 1.00 |  | 1.00 | 1.00 | 1.00 | 1.00 | 0.54 | 1.00 | 1.00 |
| ***Trifolium pratense*** | 1.00 | 1.00 | 1.00 |  | 1.00 | 1.00 | 1.00 | 1.00 | 1.00 | 0.72 |
| ***Jasione montana*** | 1.00 | 1.00 | 1.00 | 1.00 |  | 1.00 | 1.00 | 1.00 | 1.00 | 0.89 |
| ***Centaurea jacea*** | 1.00 | 1.00 | 1.00 | 1.00 | 1.00 |  | 1.00 | 0.20 | 1.00 | 1.00 |
| ***Berteroa incana*** | 1.00 | 1.00 | 1.00 | 1.00 | 1.00 | 1.00 |  | 0.11 | 0.70 | 1.00 |
| ***Centaurea stoebe*** | 0.98 | 0.02 | 0.54 | 1.00 | 1.00 | 0.20 | 0.11 |  | 1.00 | **0.00** |
| ***Origanum vulgare*** | 1.00 | 0.14 | 1.00 | 1.00 | 1.00 | 1.00 | 0.70 | 1.00 |  | **0.02** |
| ***Solidago gigantea*** | 0.79 | 1.00 | 1.00 | 0.72 | 0.89 | 1.00 | 1.00 | **0.00** | **0.02** |  |
|  | | | | | | | | | | |
|  | | | | | | | | | | |
| **N (p)** | ***Cirsium arvense*** | ***Echium vulgare*** | ***Senecio jacobaea*** | ***Trifolium pratense*** | ***Jasione montana*** | ***Centaurea jacea*** | ***Berteroa incana*** | ***Centaurea stoebe*** | ***Origanum vulgare*** | ***Solidago gigantea*** |
| ***Cirsium arvense*** |  | 1.00 | 1.00 | 1.00 | 1.00 | 1.00 | 1.00 | 0.88 | 1.00 | 1.00 |
| ***Echium vulgare*** | 1.00 |  | 1.00 | 1.00 | 1.00 | 1.00 | 1.00 | **0.01** | 1.00 | 1.00 |
| ***Senecio jacobaea*** | 1.00 | 1.00 |  | 1.00 | 1.00 | 1.00 | 1.00 | 0.52 | 1.00 | 1.00 |
| ***Trifolium pratense*** | 1.00 | 1.00 | 1.00 |  | 1.00 | 1.00 | 1.00 | 0.82 | 1.00 | 1.00 |
| ***Jasione montana*** | 1.00 | 1.00 | 1.00 | 1.00 |  | 1.00 | 1.00 | 0.57 | 1.00 | 1.00 |
| ***Centaurea jacea*** | 1.00 | 1.00 | 1.00 | 1.00 | 1.00 |  | 1.00 | 0.25 | 1.00 | 1.00 |
| ***Berteroa incana*** | 1.00 | 1.00 | 1.00 | 1.00 | 1.00 | 1.00 |  | 0.48 | 1.00 | 1.00 |
| ***Centaurea stoebe*** | 0.88 | **0.01** | 0.52 | 0.82 | 0.57 | 0.25 | 0.48 |  | 1.00 | **0.00** |
| ***Origanum vulgare*** | 1.00 | 1.00 | 1.00 | 1.00 | 1.00 | 1.00 | 1.00 | 1.00 |  | 0.63 |
| ***Solidago gigantea*** | 1.00 | 1.00 | 1.00 | 1.00 | 1.00 | 1.00 | 1.00 | **0.00** | 0.63 |  |
|  |  |  |  |  |  |  |  |  |  |  |
|  | | | | | | | | | | |
| **H (p)** | ***Cirsium arvense*** | ***Echium vulgare*** | ***Senecio jacobaea*** | ***Trifolium pratense*** | ***Jasione montana*** | ***Centaurea jacea*** | ***Berteroa incana*** | ***Centaurea stoebe*** | ***Origanum vulgare*** | ***Solidago gigantea*** |
| ***Cirsium arvense*** |  | 1.00 | 1.00 | 1.00 | 1.00 | 1.00 | 1.00 | 0.88 | 1.00 | 1.00 |
| ***Echium vulgare*** | 1.00 |  | 1.00 | 1.00 | 1.00 | 1.00 | 1.00 | **0.01** | 1.00 | 1.00 |
| ***Senecio jacobaea*** | 1.00 | 1.00 |  | 1.00 | 1.00 | 1.00 | 1.00 | 0.52 | 1.00 | 1.00 |
| ***Trifolium pratense*** | 1.00 | 1.00 | 1.00 |  | 1.00 | 1.00 | 1.00 | 0.82 | 1.00 | 1.00 |
| ***Jasione montana*** | 1.00 | 1.00 | 1.00 | 1.00 |  | 1.00 | 1.00 | 0.57 | 1.00 | 1.00 |
| ***Centaurea jacea*** | 1.00 | 1.00 | 1.00 | 1.00 | 1.00 |  | 1.00 | 0.25 | 1.00 | 1.00 |
| ***Berteroa incana*** | 1.00 | 1.00 | 1.00 | 1.00 | 1.00 | 1.00 |  | 0.48 | 1.00 | 1.00 |
| ***Centaurea stoebe*** | 0.88 | **0.01** | 0.52 | 0.82 | 0.57 | 0.25 | 0.48 |  | 1.00 | **0.00** |
| ***Origanum vulgare*** | 1.00 | 1.00 | 1.00 | 1.00 | 1.00 | 1.00 | 1.00 | 1.00 |  | 0.63 |
| ***Solidago gigantea*** | 1.00 | 1.00 | 1.00 | 1.00 | 1.00 | 1.00 | 1.00 | **0.00** | 0.63 |  |
