# Supplementary material for: Blooming Urban Table: Flower Resources for Butterflies in Small Wastelands of a Large European City
Source: Ecol Evol. 2025 Sep 15;15(9):e72088. doi: 10.1002/ece3.72088 (PMC12434404; doi:10.1002/ece3.72088)
Supplement: Supplementary file 6 — Appendix S6: ece372088‐sup‐0001‐AppendixS6.docx. [file ECE3-15-e72088-s004.docx]

Appendix 6. Number of butterfly species and individuals recorded on each flowering plant during quantitative analysis

***Berteroa incana***

| Sample number | Bi1 | Bi2 | Bi3 | Bi4 | Bi5 | Bi6 | Bi7 | Bi8 | Bi9 | Bi10 |
| --- | --- | --- | --- | --- | --- | --- | --- | --- | --- | --- |
| *Pieris brassicae* | 0 | 0 | 0 | 1 | 0 | 0 | 0 | 0 | 0 | 0 |
| *Polyommatus coridon* | 2 | 1 | 3 | 4 | 0 | 0 | 0 | 0 | 0 | 0 |
| *Thymelicus lineola* | 0 | 1 | 1 | 0 | 0 | 0 | 0 | 0 | 0 | 0 |
| *Pieris rapae* | 0 | 0 | 0 | 1 | 1 | 0 | 0 | 0 | 0 | 0 |
| *Pontia edusa* | 1 | 0 | 2 | 2 | 0 | 0 | 0 | 0 | 0 | 0 |
| *Lycaena tityrus* | 0 | 0 | 0 | 1 | 0 | 0 | 0 | 0 | 0 | 0 |
| *Lycaena phlaeas* | 1 | 1 | 0 | 0 | 0 | 0 | 0 | 1 | 1 | 0 |
| *Aricia agestis* | 0 | 0 | 0 | 2 | 0 | 0 | 0 | 0 | 1 | 0 |
| *Polyommatus icarus* | 0 | 0 | 0 | 2 | 3 | 0 | 0 | 1 | 1 | 0 |
| *Inachis io* | 0 | 0 | 0 | 0 | 14 | 0 | 0 | 0 | 0 | 0 |
| *Maniola jurtina* | 0 | 0 | 0 | 0 | 10 | 0 | 0 | 0 | 18 | 0 |

***Jasione montana***

| Sample number | Jm1 | Jm2 | Jm3 | Jm4 | Jm5 | Jm6 | Jm7 | Jm8 | Jm9 | Jm10 | Jm11 |
| --- | --- | --- | --- | --- | --- | --- | --- | --- | --- | --- | --- |
| *Polyommatus icarus* | 0 | 0 | 0 | 0 | 1 | 9 | 0 | 5 | 7 | 0 | 0 |
| *Polyommatus coridon* | 0 | 0 | 0 | 0 | 39 | 24 | 14 | 28 | 0 | 8 | 9 |
| *Thymelicus lineola* | 0 | 0 | 0 | 0 | 1 | 0 | 1 | 1 | 1 | 0 | 0 |
| *Maniola jurtina* | 6 | 0 | 5 | 3 | 0 | 0 | 1 | 1 | 16 | 9 | 0 |
| *Aphantophus hiperantus* | 0 | 0 | 0 | 0 | 0 | 0 | 0 | 0 | 15 | 9 | 1 |
| *Pontia edusa* | 0 | 0 | 0 | 0 | 0 | 0 | 0 | 2 | 0 | 0 | 0 |
| *Vanessa cardui* | 0 | 0 | 10 | 0 | 0 | 0 | 0 | 0 | 0 | 0 | 0 |
| *Aricia agestis* | 0 | 0 | 0 | 0 | 3 | 0 | 1 | 3 | 0 | 2 | 0 |
| *Lycaena tityrus* | 0 | 0 | 0 | 0 | 2 | 0 | 0 | 0 | 5 | 3 | 0 |
| *Lycaena phlaeas* | 0 | 0 | 0 | 0 | 0 | 0 | 1 | 0 | 0 | 0 | 0 |
| *Hyponephele likaon* | 0 | 0 | 0 | 0 | 0 | 0 | 0 | 2 | 0 | 0 | 0 |
| *Coenonympha pamphilus* | 0 | 0 | 0 | 0 | 3 | 3 | 1 | 3 | 0 | 0 | 0 |
| *Pieris rapae* | 0 | 0 | 0 | 0 | 1 | 0 | 0 | 0 | 0 | 0 | 0 |
| *Issoria lathonia* | 0 | 0 | 0 | 0 | 1 | 0 | 0 | 0 | 0 | 0 | 0 |
| *Erynnis tages* | 0 | 0 | 0 | 0 | 0 | 2 | 0 | 0 | 0 | 0 | 0 |

***Echium vulgare***

| Sample number | Ev1 | Ev2 | Ev3 | Ev4 | Ev5 | Ev6 | Ev7 | Ev8 |
| --- | --- | --- | --- | --- | --- | --- | --- | --- |
| *Thymelicus lineola* | 0 | 0 | 1 | 2 | 2 | 13 | 22 | 0 |
| *Pieris rapae* | 0 | 0 | 0 | 0 | 0 | 0 | 0 | 3 |
| *Ochlodes sylvanus* | 0 | 0 | 20 | 25 | 22 | 0 | 0 | 28 |
| *Polyommatus coridon* | 0 | 0 | 0 | 0 | 0 | 0 | 4 | 0 |
| *Erynnis tages* | 0 | 0 | 0 | 0 | 0 | 7 | 2 | 0 |
| *Polyommatus icarus* | 0 | 0 | 0 | 0 | 0 | 1 | 0 | 0 |
| *Coenonympha pamphilus* | 0 | 0 | 0 | 0 | 0 | 0 | 0 | 6 |

***Centaurea jacea***

| Sample number | Cj1 | Cj2 | Cj3 | Cj4 | Cj5 | Cj6 | Cj7 | Cj8 | Cj9 | Cj10 |
| --- | --- | --- | --- | --- | --- | --- | --- | --- | --- | --- |
| *Melanargia galanthea* | 1 | 1 | 0 | 6 | 1 | 1 | 8 | 12 | 1 | 0 |
| *Maniola jurtina* | 19 | 10 | 1 | 1 | 0 | 3 | 2 | 1 | 3 | 0 |
| *Polyommatus icarus* | 1 | 1 | 0 | 3 | 3 | 0 | 0 | 0 | 0 | 1 |
| *Inachis io* | 0 | 2 | 0 | 1 | 4 | 0 | 0 | 0 | 0 | 0 |
| *Pieris brasicace* | 0 | 0 | 1 | 0 | 0 | 0 | 0 | 0 | 0 | 0 |
| *Pieris rapae* | 0 | 0 | 0 | 2 | 0 | 0 | 1 | 0 | 0 | 0 |
| *Aphantopus hyperantus* | 0 | 0 | 0 | 0 | 0 | 1 | 0 | 0 | 0 | 0 |

***Centaurea stoebe***

| Sample number | Cs1 | Cs2 | Cs3 | Cs4 | Cs5 | Cs6 | Cs7 |
| --- | --- | --- | --- | --- | --- | --- | --- |
| *Aphantopus hyperantus* | 6 | 9 | 0 | 0 | 0 | 1 | 0 |
| *Aricia agestis* | 3 | 0 | 6 | 11 | 0 | 4 | 0 |
| *Boloria dia* | 0 | 0 | 0 | 0 | 0 | 1 | 0 |
| *Erynnis tages* | 0 | 0 | 0 | 0 | 0 | 0 | 3 |
| *Maniola jurtina* | 78 | 73 | 75 | 59 | 89 | 47 | 3 |
| *Melanarghia galanthea* | 110 | 160 | 45 | 28 | 38 | 67 | 6 |
| *Ochlodes sylvanus* | 5 | 2 | 9 | 7 | 9 | 0 | 0 |
| *Pieris brasicace* | 0 | 0 | 0 | 0 | 1 | 0 | 0 |
| *Pieris napi* | 1 | 0 | 8 | 0 | 6 | 4 | 0 |
| *Pieris rapae* | 1 | 0 | 0 | 0 | 1 | 0 | 0 |
| *Polygonia c-album* | 2 | 3 | 0 | 0 | 0 | 0 | 0 |
| *Polyommatus icarus* | 1 | 0 | 0 | 0 | 0 | 0 | 2 |
| *Thymelicus lineola* | 88 | 54 | 45 | 43 | 67 | 79 | 0 |
| *Vanessa cardui* | 6 | 5 | 0 | 0 | 0 | 0 | 0 |

***Solidago gigantea***

| Sample number | Sg1 | Sg2 | Sg3 | Sg4 | Sg5 | Sg6 | Sg7 |
| --- | --- | --- | --- | --- | --- | --- | --- |
| *Lycaena phlaeas* | 0 | 1 | 0 | 0 | 1 | 1 | 0 |
| *Polyommatus icarus* | 0 | 0 | 0 | 1 | 0 | 0 | 0 |
| *Pieris napi* | 0 | 0 | 0 | 0 | 1 | 0 | 0 |
| *Aricia agestis* | 0 | 0 | 0 | 1 | 0 | 0 | 0 |
| *Inachis io* | 0 | 0 | 0 | 0 | 1 | 0 | 12 |

***Cirsium arvense***

| Sample number | Ca1 | Ca2 | Ca3 | Ca4 | Ca5 | Ca6 | Ca7 | Ca8 | Ca9 | Ca10 | Ca11 | Ca12 | Ca13 |
| --- | --- | --- | --- | --- | --- | --- | --- | --- | --- | --- | --- | --- | --- |
| *Gonepteryx rhamni* | 0 | 0 | 0 | 0 | 0 | 1 | 0 | 0 | 0 | 0 | 0 | 0 | 0 |
| *Inachis io* | 10 | 20 | 6 | 9 | 3 | 24 | 7 | 1 | 0 | 0 | 0 | 0 | 0 |
| *Lycaena tityrus* | 0 | 0 | 0 | 0 | 0 | 0 | 0 | 0 | 0 | 0 | 0 | 0 | 0 |
| *Maniola jurtina* | 23 | 51 | 8 | 13 | 14 | 26 | 3 | 5 | 3 | 3 | 8 | 19 | 13 |
| *Pieris brassicae* | 0 | 1 | 0 | 0 | 0 | 0 | 0 | 0 | 0 | 0 | 0 | 0 | 0 |
| *Pieris napi* | 1 | 0 | 0 | 0 | 0 | 0 | 0 | 0 | 0 | 0 | 0 | 0 | 0 |
| *Pontia edusa* | 0 | 0 | 0 | 0 | 0 | 0 | 0 | 0 | 1 | 0 | 0 | 0 | 0 |
| *Thymelicus lineola* | 1 | 0 | 0 | 0 | 0 | 0 | 0 | 0 | 0 | 0 | 0 | 0 | 0 |
| *Melanargia galanthea* | 3 | 2 | 4 | 5 | 2 | 2 | 0 | 0 | 3 | 0 | 1 | 1 | 1 |
| *Pieris rapae* | 4 | 13 | 2 | 0 | 1 | 1 | 0 | 0 | 0 | 0 | 0 | 0 | 0 |
| *Aphantopus hyperantus* | 1 | 0 | 6 | 4 | 0 | 0 | 0 | 0 | 0 | 0 | 1 | 0 | 0 |
| *Lycaena phlaeas* | 0 | 0 | 3 | 0 | 1 | 0 | 0 | 0 | 0 | 0 | 0 | 0 | 0 |

***Trifolium pratense***

| Sample number | Tp1 | Tp2 | Tp3 | Tp4 | Tp5 | Tp6 | Tp7 | Tp8 | Tp9 |
| --- | --- | --- | --- | --- | --- | --- | --- | --- | --- |
| *Cupido argiades* | 0 | 0 | 1 | 0 | 0 | 0 | 0 | 0 | 0 |
| *Gonepteryx rhamni* | 5 | 16 | 6 | 4 | 8 | 0 | 0 | 0 | 0 |
| *Inachis io* | 9 | 4 | 5 | 25 | 32 | 0 | 0 | 0 | 0 |
| *Melanarghia galanthea* | 2 | 2 | 1 | 1 | 1 | 0 | 0 | 0 | 0 |
| *Pieris brassicae* | 1 | 1 | 0 | 2 | 2 | 1 | 0 | 0 | 0 |
| *Pieris rapae* | 0 | 0 | 2 | 0 | 0 | 0 | 0 | 0 | 0 |
| *Polyommatus icarus* | 0 | 0 | 0 | 0 | 0 | 0 | 0 | 1 | 0 |
| *Thymelicus lineola* | 1 | 1 | 6 | 1 | 2 | 0 | 0 | 0 | 0 |
| *Vanessa cardui* | 1 | 1 | 2 | 0 | 8 | 0 | 0 | 0 | 0 |
| *Maniola jurtina* | 1 | 0 | 1 | 1 | 2 | 0 | 0 | 0 | 0 |
| *Ochlodes sylvanus* | 0 | 0 | 0 | 0 | 0 | 0 | 0 | 0 | 1 |

***Senecio jacobaea***

| Sample number | Sj1 | Sj2 | Sj3 | Sj4 | Sj5 | Sj6 | Sj7 | Sj8 | Sj9 | Sj10 | Sj11 | Sj12 |
| --- | --- | --- | --- | --- | --- | --- | --- | --- | --- | --- | --- | --- |
| *Maniola jurtina* | 0 | 0 | 1 | 27 | 29 | 36 | 25 | 10 | 12 | 0 | 5 | 9 |
| *Araschnia levana* | 0 | 0 | 0 | 0 | 12 | 12 | 7 | 12 | 8 | 6 | 2 | 14 |
| *Aphantopus hyperantus* | 0 | 0 | 0 | 17 | 12 | 18 | 99 | 80 | 89 | 5 | 2 | 5 |
| *Lycaena phlaeas* | 0 | 0 | 0 | 3 | 0 | 0 | 1 | 0 | 0 | 0 | 0 | 0 |
| *Pieris napi* | 0 | 0 | 0 | 0 | 0 | 2 | 0 | 1 | 0 | 0 | 0 | 0 |
| *Celastrina argiolus* | 0 | 0 | 0 | 0 | 0 | 1 | 0 | 0 | 0 | 0 | 0 | 0 |
| *Melanarghia galanthea* | 0 | 0 | 0 | 0 | 0 | 1 | 0 | 0 | 0 | 0 | 0 | 4 |
| *Thymelicus lineola* | 0 | 0 | 0 | 0 | 0 | 0 | 1 | 0 | 0 | 2 | 0 | 1 |
| *Polygonia c-album* | 0 | 0 | 0 | 0 | 0 | 0 | 0 | 0 | 1 | 0 | 2 | 2 |

***Origanum vulgare***

| Sample number | Ov1 | Ov2 | Ov3 | Ov4 | Ov5 | Ov6 | Ov7 |
| --- | --- | --- | --- | --- | --- | --- | --- |
| *Aphantopus hyperantus* | 47 | 31 | 16 | 24 | 12 | 17 | 11 |
| *Inachis io* | 1 | 0 | 2 | 0 | 2 | 0 | 1 |
| *Issoria lathonia* | 2 | 0 | 0 | 0 | 0 | 14 | 0 |
| *Melanarghia galanthea* | 3 | 1 | 0 | 1 | 1 | 2 | 0 |
| *Pieris napi* | 0 | 1 | 1 | 0 | 1 | 0 | 0 |
| *Pieris brasicace* | 0 | 0 | 1 | 0 | 0 | 0 | 0 |
| *Pieris rapae* | 3 | 3 | 1 | 0 | 0 | 1 | 0 |
| *Aricia agestis* | 0 | 0 | 0 | 0 | 1 | 0 | 0 |
| *Araschnia levana* | 0 | 0 | 0 | 0 | 0 | 2 | 16 |
| *Aglais urticae* | 0 | 0 | 0 | 0 | 0 | 1 | 0 |
| *Maniola jurtina* | 7 | 1 | 6 | 6 | 3 | 2 | 0 |
| *Ochlodes sylvanus* | 0 | 0 | 1 | 1 | 0 | 0 | 0 |
